# *Intra-vitam* Diagnosis of Tuberculosis in Pigs: Concordance Between Interferon-Gamma Release Assay and Comparative Tuberculin Skin Test

**DOI:** 10.3389/fvets.2020.591444

**Published:** 2020-12-18

**Authors:** Dorotea Ippolito, Michele Fiasconaro, Flavia Pruiti Ciarello, Antonino Aliberti, Maria Vitale, Benedetta Amato, Paolo Pasquali, Vincenzo Di Marco Lo Presti

**Affiliations:** ^1^Istituo Zooprofilattico Sperimentale della Sicilia “A. Mirri”, Area Territoriale Barcellona Pozzo di Gotto, Messina, Italy; ^2^Veterinary Practitioner, Messina, Italy; ^3^Department of Food Safety, Nutrition and Veterinary Public Health, Istituto Superiore di Sanità, Rome, Italy

**Keywords:** tuberculosis, *M. bovis*, pig, tuberculin skin test, interferon-gamma, *intra-vitam* diagnosis

## Abstract

The role of pigs in the maintenance of bovine tuberculosis caused by *Mycobacterium bovis* has been demonstrated in many settings; however, the current control programs usually do not state any *intra-vitam* diagnostic procedure in this species, as for the cattle. Carcass inspection has shown to be insufficient to detect infection in swine; thus, the assessment of intradermal tuberculin test and interferon-gamma release assay (IGRA) in this species is mandatory. The current study compares the performances of the single intradermal comparative cervical tuberculin (SICCT) test and IGRA. A total of 628 Nebrodi Black pigs raised in free-roaming farms were subjected to the two tests simultaneously. Besides, 124 animals underwent *postmortem* examination for the detection of tuberculous lesions and isolation of mycobacteria from target organs. The two tests showed a concordance of 94.42% with a Cohen's k coefficient of 0.786 and McNemar chi-square of 4.83 (*P* = 0.03). Slightly lower levels of concordance (90.32%) between SICCT and IGRA were obtained in the group of 124 animals, with a Cohen's *k* = 0.797 and McNemar chi-squared value of 0.69 with a non-significant *P* = 0.41. Moreover, the results showed how IGRA tends to result positive in higher rates, mostly when non-tuberculous mycobacteria (NTM) were isolated, suggesting a possible impairment of specificity in the event of coinfections in the swine. In conclusion, the results obtained support the possibility of the strategic use of IGRA or SICCT in combination or alternatively one to the other, particularly IGRA which showed lower specificity but has evident advantages over SICCT.

## Introduction

Despite being named after its main host, bovine tuberculosis (bTB) is a chronic, weakening infectious disease caused by *Mycobacterium bovis* which infects a broad host spectrum (1). The disease, mainly detected in cattle, can also affect various domestic species including goats, sheep, camelids, buffaloes, and domestic carnivores, along with several wildlife species. The Suidae family has been demonstrated to be highly susceptible to mycobacteriosis caused by *M. bovis*, and the detection of severe generalized lesions reported in pigs and wild boars suggests that those species may act as maintenance hosts rather than dead-end hosts in peculiar epidemiological contexts (2, 3). *M. bovis* infections are more rarely encountered in settings were bTB eradication programs are highly effective and the swine industry is mainly based on intensive rational breeding farms. On the contrary, bTB seems to occur mainly in areas where pigs are bred in free-roaming systems and where the density of infected wildlife (e.g., wild boars) is high, with or without the presence of bovines (4). The existence of a dynamic interface between environment, livestock, and wildlife is highlighted as well by the presence of several *M. bovis* strains shared between species, especially in those settings with free-roaming animals sharing the same environment (5). The role of wildlife seems to be crucial for the disease persistence (6) as it is well-established that sharing pastures and water sources between infected and uninfected animals makes the interspecies transmission very likely (7). Thus, in this scenario the application of systematic *intra-vitam* testing restricted to cattle and neglecting the role of the other susceptible species poses several obstacles to the current eradication programs in many countries. The two available *intra-vitam* assays are the single intradermal tuberculin (SIT) test and interferon-gamma release assay (IGRA), which measure the cell-mediated immune response (CMI), the earliest response to the infection (8). These tests have been adapted from the bovine species and the scientific literature dealing with their use in suids is scarce. There are indeed few reports on farmed pigs (9, 10) and some reports on wild boards (11). In particular, Jaroso et al. (11) in 2010 suggested the potential use of Single Intradermal Comparative Cervical Tuberculin (SICCT) test for the diagnosis of bTB in Eurasian wild boards. The study, however, highlighted the difficulties related to the restraint of the animals that make this test scarcely applicable to the routine bTB testing in the field. Conversely, in a previous study (10), our group already tested IGRA performances on pigs. The study showed that IGRA is an easy-to-apply test, requiring just a blood sampling, and suggested the possible large-scale use of IGRA in pigs, due to the high diagnostic power and the reliable accuracy (10). Similarly, tests measuring the humoral immune response on sera samples have been considered as convenient tools to evaluate the prevalence of bTB in suids (12–14). However, to the best of our knowledge, extensive data on the application of these methods still lack in farmed pigs. The primary purpose of the present study is to compare the performances of the two *intra-vitam* diagnostic methods (IGRA and SICCT), in support of future decisions concerning bTB control programs in pigs.

## Materials and Methods

The area under study is the Nebrodi Natural Park Area, sited northwest in the island of Sicily, Italy. The Park is characterized by an extensive farming system, where different species (e.g., cattle, sheep, goats, horses, and donkeys) are often bred in promiscuity. Moreover, contacts between livestock and wildlife (e.g., feral pigs, wild boars, deers, foxes, martens, porcupines) are not uncommon, due to shared pastures and watering areas. Among the porcine breeds, one of the most common is the endogenous Nebrodi Black Pig, a small-size dark-coated pig which resembles the wild boar, due to the overlapping habitat occupied, and the similar social, behavioral and alimentary habits. Animals kept in 45 free-range farms were included, according to the willingness of the breeders to participate in the study. All the breeding pigs present within the farms (for a total number of 628 animals) were tested both by SICCT and IGRA. According to the need of renewing old breeders, 124 subjects were slaughtered during the study period and were subjected to *postmortem* examination (PME) at the abattoir.

### Comparative Tuberculin Skin Test

The Single Intradermal Comparative Cervical Tuberculin (SICCT) test was carried out after the appropriate individual restraint of each animal. The test was performed injecting intradermally the basal area of both the auricular pinnae with 0.1 mL of bovine purified protein derivative (b-PPD, 5,000 UI) and 0.2 mL of avian PPD (a-PPD, 5,000 UI), obtained from Istituto Zooprofilattico Sperimentale dell' Umbria e delle Marche (IZSUM, Perugia, Italy). Skin folds were measured after 72 h by the use of a caliper, and interpretation of the results was carried out according to the legislation in force (European Union Directive 97/12/EC), applied to cattle, as follows:

Positive reactor: a positive b-PPD reaction which is more than 4 mm greater than the a-PPD reaction, or the presence of clinical signs;

Inconclusive: a positive or inconclusive b-PPD reaction which is from 2 to 4 mm greater than the a-PPD reaction, and the absence of clinical signs;

Negative reactor: a negative b-PPD reaction, or a positive or inconclusive b-PPD reaction but which is equal to or less than a positive or inconclusive a-PPD reaction and the absence of clinical signs in both cases.

### Interferon-Gamma Release Assay

Interferon-gamma (IFNγ) release assay (IGRA) was performed according to Pesciaroli et al. (10) on whole blood samples collected in heparinized tubes by venipuncture from each animal before SICCT was performed. Four aliquots of blood (1.5 mL) were separately stimulated in a 24-flat-bottomed-well plate with a-PPD (10 μg/mL), b-PPD (10 μg/mL), phosphate buffer saline (PBS) as negative control and pokeweed mitogen (PWM, Sigma Aldrich) as a positive control, to assess lymphocytes vitality and responsiveness. Incubation was performed overnight at 37° and 5% CO2.

Supernatants obtained were then collected and processed using a commercial kit (Quantikine Elisa kit, Porcine IFNγ, R&D systems) according to the manufacturer's instructions. Plates were read using a spectrophotometer (Multiskan FC 51119000, Thermo Scientific) at a 450-nm wavelength, and results were expressed as optical densities (OD). Interpretation of the results was performed according to Cagiola et al. (15), using the following criteria:

Unsuitable: OD (PBS) > 0.15;

*M. bovis* reactor if: OD (b-PPD) ≥ 2 OD (PBS) or OD (b-PPD)/ OD (a-PPD) ≥ 1.1;

*M. avium* reactor if OD (a-PPD) ≥ 2 OD (PBS) or OD (b-PPD)/ OD (a-PPD) ≤ 0.9;

Unresolved if OD (b-PPD)/ OD (a-PPD) between 0.9 and 1.1.

### Anatomopathological Examination

The *postmortem* examination (PME) was performed by the systematic inspection at the slaughterhouse. Each carcass was sectioned, and target organs were examined. Particularly, lymph nodes of the head region (retropharyngeal, submandibular, parotidean and the tonsils), the thorax (bronchoalveolar and mediastinal), and the abdomen (mesenteric), as well as the heart and lungs were inspected and collected for further analysis. Abdominal organs (spleen, liver, and the gastrointestinal tract) were also inspected and collected.

### *M. bovis* Isolation and Identification

Tissue samples were collected from organs abovementioned and processed according to the standard operating procedures, approved by the Italian certifying body ACCREDIA, in use in the Laboratory of Istituto Zooprofilattico Sperimentale della Sicilia (IZS-Barcellona Pozzo di Gotto, Italy) (16). Tissue homogenates were decontaminated using NaOH 1 N and then neutralized. After several steps of washing and centrifugation, the sediment was cultured using the liquid culture system BACTEC-MGIT 960 (Becton Dickinson) according to the manufacturer's instructions. Incubation was performed at 37°C ± 2. Samples showing no growth within 6 weeks were considered negative. Conversely, if any growth was evident, an aliquot of the sample was smeared onto a slide and submitted to Ziehl–Neelsen staining to confirm the presence of mycobacteria and to exclude eventual contamination. If any contamination was present, the vial containing the isolate underwent to an additional decontamination step and then incubated again in the BACTEC-MGIT 960 system. All the positive samples were sent to the National Reference Center for Tuberculosis, Istituto Zooprofilattico Sperimentale della Lombardia e dell' Emilia Romagna (IZSLER-Brescia, Italy). The isolates were submitted to PCR for the detection of microorganisms belonging to the genus *Mycobacterium* spp., and the mycobacterium tuberculosis complex (MTBC) according to the accredited procedure in use (MP 09/004, rev. 2/2012). In the case of positivity, high-resolution melt (HRM) analysis targeting the gyrB gene was performed to assess the presence of *M. bovis*. In the case of *Mycobacterium* spp. positive samples resulted negative to MTBC identification, infection by non-tuberculous mycobacteria (NTM) was diagnosed.

### Statistical Analysis

To compare the performance of the diagnostic assays, results were examined in contingency tables and concordance test and Cohen's k coefficient (k) were used. McNemar's test was used to assess any difference in the performance of the two tests. Significance was set for a *p*-value (*P*) ≤ 0.5.

## Results

### *Intra-vitam* Diagnostic Approach

Data on the performance of SICCT and IGRA were available for 628 animals, testing positive in 90 (14.33%) and 103 (16.4%) animals, respectively (Table 1). When the performances of the two tests were compared, concordance accounted for 94.42%, with a McNemar chi-squared = 4.83 (*P* = 0.03) and *k* = 0.786 (good), demonstrating how these two tests are highly comparable in the context examined. Within this group, 79 animals tested positive for both tests, 11 animals for SICCT and 24 animals for IGRA.

**Table 1:** Total animals tested using IGRA and SICCT.

	**IGRA+**	**IGRA−**	**Total**
SICCT+	79	11	90
SICCT−	24	514	538
**Total**	**103**	**525**	**628**

### Comparison of *Intra-vitam* Diagnosis With Microbiological and *Postmortem* Analysis

#### Mycobacteria Isolation and Identification

According to the microbiological and molecular analysis performed on the 124 carcasses submitted to PME, the animals were grouped as follows:

*M. bovis* positive group (21/124, 16.9%) (Table 2). SICCT and IGRA gave positive results in 19 (90.5%) and negative results in 2 (9.5%) animals, respectively (Table 2), with 100% concordance and *k* = 1.

**Table 2:** Analysis performed in *M. bovis* culture positive animals.

	**IGRA+**	**IGRA−**	**Total**
SICCT+	19	0	19
SICCT−	0	2	2
**Total**	**19**	**2**	**21**

Non-tuberculous mycobacteria (NTM) positive group (30/124 = 24.2%) (Table 3). SICCT and IGRA gave positive results in 9 (30.0%) and 14 (46.7%) animals, respectively. Concordance was moderate (*k* = 0.521), McNemar chi-square was 3.57 (*P* = 0.06). Within this group, eight animals tested positive for both tests, one animal for SICCT and six animals for IGRA.

**Table 3:** Analysis performed on non-tuberculous mycobacteria (NTM) positive animals.

	IGRA+	IGRA−	Total
SICCT+	8	1	9
SICCT−	6	15	21
**Total**	**14**	**16**	**30**

Culture negative group (73/124, 58.9%) (Table 4). SICCT and IGRA gave positive results in 17 (20.5%) and 15 (23.3%) animals, respectively. Concordance was good (*k* = 0.760), McNemar chi-square statistic was 0.67 (*P* = 0.41). Within this group, 13 animals tested positive for both tests, four animals for SICCT and two animals for IGRA.

**Table 4:** Analysis performed on culture-negative animals.

	IGRA+	IGRA−	Total
SICCT+	13	4	17
SICCT−	2	54	56
**Total**	**15**	**58**	**73**

#### Lesions

**Figure 1:**
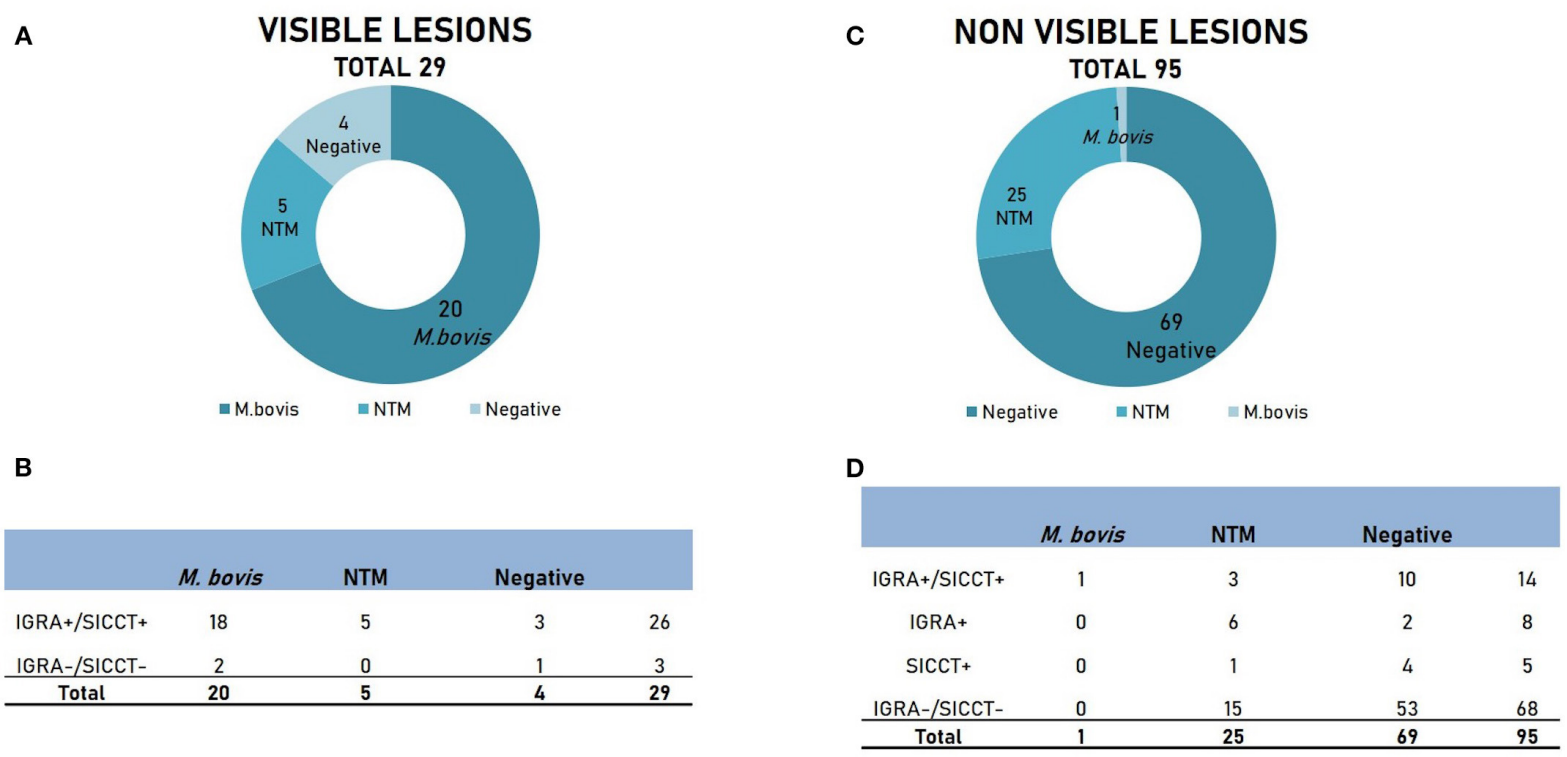
Results of culture and *intra-vitam* assays of animals showing visible lesions (VLs) and non-visible lesions (NVLs). **(A)** Microbiological results in animals with VLs. **(B)** Results of *intra-vitam* assays in animals with VLs. **(C)** Microbiological results in animals with NVLs. **(D)** Results of *intra-vitam* assays in animals with NVLs.

Twenty-nine out of 124 animals (23.4%) showed visible lesions (VLs) referable to tuberculosis (Figure 1A). From these animals, *M. bovis* was isolated in 20/29 cases (69%), while NTM were identified in a smaller percentage of samples (5/29, 17.2%). Interestingly, four samples out of 29 showing VLs did not show any growth of mycobacteria (13.8%). *Intra-vitam* tests on these animals were highly concordant, with 26 animals positive and three negatives to both tests (Figure 1B). Most of the animals affected by *M. bovis* showed concordant positivity to both assays (18/20, 90%). Surprisingly, all five animals affected by NTM showed positivity to both *intra-vitam* assays. However, both IGRA and SICCT did not detect three animals out of 29 (10.3%) showing VLs, among which two were positive to *M. bovis*.

The remaining 95 animals (76.6%) did not show any visible lesions (NVLs, Figure 1C). No mycobacteria were isolated from the majority of the samples (69/95, 72.6%), while NTM were isolated in 25/95 samples (26.3%). Only one sample (1.05%) was positive to *M. bovis*. The overall response to *intra-vitam* tests is described in Figure 1D. Both culture-negative and NTM group tested largely negative to IGRA and SICCT (53/69, 76.8% and 15/25, 60%, respectively). However, a certain level of positivity to *intra-vitam* tests was recorded in both groups (16/69, 29% and 10/25, 40% for the culture-negative and the NTM group respectively). Interestingly, the only lesion-negative case positive to *M. bovis* tested positive to both IGRA and SICCT.

In conclusion, the concordance between IGRA and SICCT within the group of animals submitted to PME (124) was high, accounting for 90.32%, with a *k* = 0.797 (good) and McNemar chi-squared value of 0.69 (*P* = 0.41). Within this group, 40 animals tested positive for both tests, five animals for SICCT and eight animals for IGRA.

## Discussion

In many countries, pigs serve as dead-end spillover hosts of bTB (17, 18); however, in others, their role as maintenance hosts has been widely recognized (2, 3). Either in Spanish wild boars (2) or Nebrodi Black Pigs in Sicily (3), the high rates of reported generalized granulomatous lesions referable to bTB suggested the role of suids as maintenance hosts. Moreover, a novel strain of *M. bovis* was isolated in Nebrodi Black Pigs, raising the concern of active circulation of *M. bovis* genotypes exclusively in the porcine species (3), further corroborated by more recent studies (5).

Although bTB prevalence in swine has not been subjected to the official investigation, there is a strong body of evidence that bTB can be actively present in the pig population. This implies that the control of bTB in swine should be mandatory when the presence of those animals represents an epidemiological risk. The sole carcass inspection at the slaughterhouse, as provided for in the legislation in force, however, cannot represent the best approach. It has been shown that the isolation of *M. bovis* not always corresponds to macroscopically detectable lesions at the abattoir (19, 20) and that the most frequently affected organs in swine are mandibular lymph nodes and tonsils (21), which usually are not examined during swine carcasses official inspection. As a consequence, the control program could benefit from the application of *intra-vitam* diagnostic techniques in swine.

Overall, our investigation on the use of *intra-vitam* diagnostic techniques shows that IGRA and SICCT can detect bTB in a quite similar manner, giving high concordant results. However, despite the equal performance, IGRA tended to retrieve higher positivity rates. Twenty-four out of 628 (3.8%) animals were positive to the sole IGRA, while 11/628 (1.75%) were positive only to SICCT (Table 1). Although the only data available on pigs reported an IGRA specificity of 100% (10), the ability of other mycobacteria to interfere with *intra-vitam* diagnostic tests has not been clarified yet. In other species, the performances of SICCT and IGRA can be influenced by the exposition to environmental mycobacteria (22–24). Our results suggest that NTM-exposed pigs could be misdiagnosed by SICCT and IGRA (Table 3 and Figures 1B,D). To further dissect this issue, it could be useful to perform studies on detection of highly specific CMI response to *M. bovis* after stimulation of pig cells with antigens which are usually not expressed by NTM (such as ESAT-6 or CFP-10) (25), as already done in cattle (25, 26).

On the other hand, a higher sensitivity of IGRA cannot be ruled out. In cattle, it has been demonstrated that IFNγ is produced during the initial phase of the mycobacterial infection while the reaction induced by SICCT appears later (8). Moreover, the higher sensitivity could also be related to the higher analytical sensitivity of IGRA, able to detect low levels of IFNγ and therefore milder immune responses.

However, it has been shown how bacteriology has its limitations (27) and that sensitivity can be improved by the parallel use of PCR on organs and culture for the diagnosis of bTB in swine (28). This could explain the positivity of *intra-vitam* tests in culture-negative animals with or without VLs. We found highly concordant positivity to IGRA and SICCT (Figures 1B,D) in culture-negative animals (10.3% showing VLs and 10.5% with NVLs). Unfortunately, no PCR on tissues was performed in our study, so the supposed lack of sensitivity of microbiological examination cannot be ruled out. However, although culture and PCR are widely accepted as a gold-standard diagnostic method for bTB, Bayesian latent class statistical analysis has been recently used to assess sensitivity and specificity of the different diagnostic tools for bTB (29–34) in the absence of a “perfect” gold-standard method. Since these studies were conducted on Tuberculosis affecting cattle, similar studies are envisaged also in the pig population.

Nevertheless, because the study design is not possible to formulate any consideration on test performance; larger-scale studies performed on animals with known health status (exposed vs. non-exposed to bTB) are needed to demonstrate the hypothesis of a lower specificity of IGRA than SICCT, in swine.

Other approaches to bTB diagnosis in pigs have considered humoral response parameters in pigs (12–14). The humoral response generally raises later during mycobacterial infection (8) but seems to have an earlier onset in pigs (12). The parallel use of CMI and serological tests is useful to detect anergic states which can be misdiagnosed by CMI tests (8). As a consequence, attempting further studies combining CMI and humoral response assessment might be of some interest to improve diagnostic techniques for bTB in swine.

The high concordance between IGRA and SICCT detected in our study opens new interesting scenarios concerning the use of IGRA, both as a parallel test to SICCT or as a stand-alone test, as already suggested for bovines (35). In many regions, the strategical use of IGRA and the intradermal tuberculin test, single or comparative, in series or parallel has been applied to optimized the control programs, in areas with different prevalence levels of tuberculosis (36). To date, the European legislation does not recognize the use of IGRA as a stand-alone test for bTB in cattle. The measurement of IFNγ levels in plasma could potentially overcome most of the difficulties related to the application of SIT, especially in those species notoriously more difficult to restrain (such as pigs) or in free-roaming farming systems. The tuberculin injection and the interpretation of the results take place in two steps, requiring the restraint of the animals twice in a short interval of time (72 h), increasing the risk of stress in the animal and resulting unpractical to both the Veterinary Service and the farmer. Conversely, IFNγ measurement in plasma needs just one capture and a blood sampling, which could be performed contextually to other samplings, as laid down in other diseases control programs (such as for classical swine fever, swine vesicular disease, and Aujeszky's diseases). Moreover, intradermal tuberculin test (single or comparative) is also strictly dependent on the capacity and experience of the operator (37), while interpretation criteria for IGRA are more objective and can be adjusted to improve sensitivity or specificity (38, 39). Nevertheless, blood samples collected for IGRA should be kept at room temperature and should be processed within 8 h (40), representing in some cases a strong limitation for its massive application in the field. However, our group developed a protocol to extend the shelf-life of the blood sample, by using interleukin-enriched maintenance media, which can preserve vitality and responsiveness of IFNγ-producing lymphocytes up to 6 days post-collection (41). The use of these maintenance media could avoid the drawbacks associated with preanalytical requirements of IGRA. Moreover, the IGRA is a more reliable test than SICCT because in laboratory conditions it is possible to use positive and negative controls (PWM and PBS) to evaluate the conservation conditions of the samples and the immunological status of the animal (42, 43).

Based on our experience and the data provided, we believe that this test can be used with a certain level of confidence in the case in which a bTB eradication program would be applied to the porcine species.

In conclusion, to the best of our knowledge, there are currently no studies comparing SICCT and IGRA performances in the porcine species, nor raising the hypothesis that the two tests might be used one in alternative to the other for the *intra-vitam* bTB diagnosis in pigs. The present study provides additional data supporting the use of *intra-vitam* diagnostic assay for bTB in the swine. Moreover, it moves forward the possibility of using the sole IGRA as a diagnostic test for bTB in pigs, in the light of the evident advantages of this technique over SICCT. In particular, it is our opinion that IGRA is a valuable approach especially in areas with high bTB prevalence due to the persistence of the main risk factors, such as promiscuity between species, shared pastures, feeding and watering areas, close contact with wildlife.

## Data Availability Statement

The original contributions generated for this study are included in the article/supplementary material, further inquiries can be directed to the corresponding author/s.

## Ethics Statement

The animal study was reviewed and approved by Organismo preposto al benessere degli animali (O.P.B.A.), Istituto Zooprofilattico Sperimentale della Sicilia A. Mirri. Written informed consent was obtained from the owners for the participation of their animals in this study.

## Author Contributions

VD conceived the presented idea and planned the study. VD, PP, and DI supervised all research. FP, BA, and AA performed the *intra-vitam* tests and provided the samples. BA and MV performed the experiments. DI, MF, and BA collected the data and performed the statistical analysis and the data interpretation. DI and FP drafted the manuscript and prepared the tables and the figures. PP and VD revised the manuscript. All authors discussed the results and approved the final version of the manuscript.

## Conflict of Interest

The authors declare that the research was conducted in the absence of any commercial or financial relationships that could be construed as a potential conflict of interest.
